# Hyperammonemia in Urinary Tract Infections

**DOI:** 10.1371/journal.pone.0136220

**Published:** 2015-08-20

**Authors:** Tsuneaki Kenzaka, Ken Kato, Akihito Kitao, Koki Kosami, Kensuke Minami, Shinsuke Yahata, Miho Fukui, Masanobu Okayama

**Affiliations:** 1 Division of General Medicine, Center for Community Medicine, Jichi Medical University School of Medicine, Shimotsuke, Japan; 2 Department of General Medicine, Toyooka Public Hospital, Toyooka, Japan; 3 Division of Community Medicine and Medical Education, Kobe University Graduate School of Medicine, Kobe, Japan; 4 Division of Community Medicine and Career Development, Kobe University Graduate School of Medicine, Kobe, Hyogo, Japan; Emory University, UNITED STATES

## Abstract

**Objectives:**

The present study investigated the incidence of hyperammonemia in urinary tract infections and explored the utility of urinary obstruction relief and antimicrobial administration to improve hyperammonemia.

**Methods:**

This was an observational study. Subjects were patients who were diagnosed with urinary tract infection and hospitalized between June 2008 and June 2009. We measured plasma ammonia levels on admission in patients who were clinically diagnosed with urinary tract infection and hospitalized. We assessed each patient's level of consciousness on admission using the Glasgow Coma Scale (GCS) and performed urine and blood cultures. We also assessed hearing prior to hospitalization using the Eastern Cooperative Oncology Group performance status (ECOG-PS). In cases with high ammonia levels on admission, plasma ammonia and GCS were measured 24 hours and 5–7 days later.

**Results:**

Sixty-seven candidates were enrolled; of these, 60 cases (89.6%) with bacterial cell counts ≥10^4^ CFU/mL were studied. Five cases (8.3%) presented with high plasma ammonia levels. Cases with hyperammonemia were significantly more likely to present with low GCS scores and urinary retention rate. All five cases received antimicrobial therapy with an indwelling bladder catheter to relieve urinary retention. The case 5 patient died shortly after admission due to complicated aspiration pneumonia; in the remaining cases, plasma ammonia levels were rapidly normalized and the level of consciousness improved.

**Conclusions:**

The occurrence of hyperammonemia in urinary tract infections is not rare. The cause of hyperammonemia is urinary retention obstruction. Therefore, along with antimicrobial administration, relief of obstruction is important for the treatment of hyperammonemia caused by this mechanism.

## Introduction

Hyperammonemia is caused by hepatic disorder/failure, gastrointestinal hemorrhage, portosystemic shunt, and vesicorectal fistula, drugs such as valproate and barbiturate, and shock [1]. Obstructive urinary tract infection with urease-producing bacteria also causes hyperammonemia [2], a rare pathologic condition whose incidence is unknown.

Treatment of hepatogenic hyperammonemia includes restriction of protein intake and the use of nonabsorbable disaccharides such as lactulose and lactitol to reduce intestinal ammonia. In contrast, there are no guidelines for standard therapy for hyperammonemia due to obstructive urinary tract infection [1], but relief of urinary retention improves disorders of consciousness and hyperammonemia [3].

The present study investigated the incidence of hyperammonemia in urinary tract infections and explored the utility of urinary obstruction relief and antimicrobial administration alone to improve hyperammonemia.

## Methods

### Study Design

This was an observational study conducted at the Department of General Medicine of Toyooka Public Hospital, Hyogo, Japan. The local ethics committee of Toyooka Public Hospital approved the study and waived the requirement for written informed consent only for participants 18 years of age or older. The ethical committee required minors (and/or their parents/guardians) to provide written informed consent. All data are available upon request; relevant data are presented here.

### Study Setting and Population

Subjects were patients who were diagnosed with urinary tract infection and hospitalized between June 2008 and June 2009. The diagnosis of urinary tract infection was based on the appendix of the fourth edition of the “Criteria for evaluation of clinical efficacy of antimicrobial agents on urinary tract infection” by the UTI Subcommittee of the Clinical Evaluation Committee, Japan Society of Chemotherapy [4]. The cases that met the following (1) to (3) conditions were enrolled as candidates; cases that met condition (4) were comprised the study subjects: (1) age ≥16 years; (2) fever ≥37.5°C or low back, flank, or kidney pain; (3) pyuria ≥5 WBCs/high power field in the microscopic urine sediment; and (4) bacterial cell counts ≥10^4^ colony-forming units (CFU)/mL.

Exclusion criteria included diagnoses of urethritis, prostatitis, or epididymitis; presence of an indwelling bladder balloon; complications of the following diseases that cause hyperammonemia: hepatic disorder/failure, gastrointestinal hemorrhage, portosystemic shunt, urea cycle disorder, or vesicorectal fistula; and treatment with valproate, barbiturate, narcotic, diuretic, or cancer chemotherapy.

### Study Protocol

We measured plasma ammonia levels on admission in patients who were clinically diagnosed with urinary tract infection and hospitalized with conditions (1) to (3) at the time of medical examination. We assessed each patient’s level of consciousness on admission using the Glasgow Coma Scale (GCS) and performed urine and blood cultures. We also assessed hearing prior to hospitalization using the Eastern Cooperative Oncology Group performance status (ECOG-PS) (Table 1). In cases with high ammonia levels on admission, plasma ammonia and GCS were measured 24 hours and 5–7 days later.

**Table 1:** Eastern Cooperative Oncology Group performance status.

Grade	Performance status
0	Asymptomatic, fully active, able to carry on all pre-disease activities without restriction
1	Lightly symptomatic, restricted in physical labor, but able to walk, carry out light labor and sedentary work, e.g., light housework, office work
2	Able to walk and care for oneself, but occasionally needs assistance, unable to carry out light labor, up and about more than 50% of waking hours
3	Capable of limited self-care, frequently needs assistance, confined to bed more than 50% of waking hours
4	Unable to carry on any self-care, needs full assistance, totally confined to bed

All patients received standard antimicrobial therapy. Empirically, patients with no or low risk factors for drug-resistant pathogens were treated with a non-antipseudomonal beta-lactam antibiotic such as ceftriaxone or cefazolin. Patients with high risk factors for drug-resistant pathogens were treated with antipseudomonal beta-lactam antibiotics such as piperacillin-tazobactam, cefepime, or meropenem, plus vancomycin for Enterococcus species in some cases. Based on culture results, patients received subsequent definitive therapy.

### Ammonia Measurements

On admission, venous blood samples were collected in heparin collection tubes and immediately used for measurement. Ammonia levels were determined using a sensitive immunolabeling assay reagent, Serotec Ammonia-L (Serotec, Hokkaido, Japan). The normal range for plasma ammonia is 12–66 μg/dL.

### Data Analysis

Baseline characteristics were compared in patients with and without hyperammonemia. Statistical significance was determined by t test or Chi-square test. All statistical analyses were performed using IBM SPSS for Windows version 22.0 (IBM Inc., New York, NY). The significance level was set at P < 0.05 for all tests.

## Results

Sixty-seven candidates were enrolled; of these, 60 cases (89.6%) with bacterial cell counts? 104 CFU/mL were studied. Table 2 shows demographic characteristics of the subjects. Subjects included 21 men and 39 women. The mean age was 76.3 ± 14.9 years (range 19 to 99 years old). ECOG-PS classed 14 cases (23.3%) as grade 0, 14 cases (23.3%) as grade 1, 11 cases (18.3%) as grade 2, eight cases (13.3%) as grade 3, and 13 cases (21.7%) as grade 4. Eighteen cases (30.0%) had a disorder of consciousness with GCS scores <14. Eight cases had urinary retention (13.3%). Twenty-three cases (38.3%) were blood culture-positive for bacteria. Five cases (8.3%) presented with high plasma ammonia levels. Cases with hyperammonemia were significantly more likely to present with low GCS scores and urinary retention rate.

**Table 2:** Demographic characteristics of the subjects.

	Cases with hyperammonemia	Cases without hyperammonemia	P value
No.	5	55	
Age*, year	84.8 ± 15.9	75.5 ± 14.7	0.643
Sex			0.649
Male	1 (20.0)	20 (36.3)	
Female	4 (80.0)	35 (63.6)	
ECOG-PS			0.208
grade 0	0 (0)	14 (25.5)	
grade 1	0 (0)	14 (25.5)	
grade 2	2 (40.0)	9 (16.3)	
grade 3	1 (20.0)	7 (12.7)	
grade 4	2 (40.0)	11 (20.0)	
Glasgow Coma Scale			0.025
scores <14.	4 (80.0)	14 (25.5)	
Positive urinary retention	5 (100.0)	3 (5.5)	< 0.001
Blood culture-positive for bacteria.	1 (20.0)	22 (40.0)	0.358

*n* (%)

* Mean ± standard deviation.

Abbreviation: ECOG-PS, Eastern Cooperative Oncology Group performance status.

Results of urine cultures are shown in Table 3. *Escherichia coli* was detected in 30 cases (50.0%), followed by *Klebsiella pneumonia* in five cases (8.3%), *Proteus mirabilis* in five cases (8.3%), and *Streptococcus agalactiae* in four cases (6.7%).

**Table 3:** Urine cultures.

Bacteria	Number of cases	Rate
*Escherichia coli*	30	50.0%
*Klebsiella pneumonia*	5	8.3%
*Enterococcus faecalis*	5	8.3%
*Proteus mirabilis*	4	6.7%
*Streptococcus agalactiae*	3	5.0%
*Staphylococcus saprophyticus*	2	3.3%
*Staphylococcus epidermidis*	2	3.3%
α-Streptococcus	2	3.3%
*Klebsiella oxytoca*	2	3.3%
*Pseudomonas aeruginosa*	1	1.7%
*Citrobacter freundii*	1	1.7%
*Enterobacter aerogenes*	1	1.7%
*Enterobacter cloacae*	1	1.7%
*Enterococcus faecium*	1	1.7%

The clinical characteristics and changes in plasma ammonia levels in cases with hyperammonemia are shown in Tables 4 and 5. Their ECOG-PS grades were 2–4 and they were not necessarily bedridden, but all had urinary retention. All five cases received antimicrobial therapy and an indwelling bladder catheter to relieve urinary retention. The case 5 patient died shortly after admission due to complicated aspiration pneumonia; in the remaining cases, plasma ammonia levels were rapidly normalized and the level of consciousness improved (cases 1–4).

**Table 4:** Clinical characteristics of five cases with high plasma ammonia levels.

Case	Age	Sex	Septic shock	Acidosis	Performance status (grade)	Urinary retention	Bacteria in urine culture	Urease production by bacteria	Bacteria in blood culture
1	61	F	No	No	3	Yes	*Escherichia coli*	No	*Escherichia coli*
2	94	M	No	No	2	Yes	α-Streptococcus	No	Not detected
3	94	F	No	No	2	Yes	*Staphylococcus epidermidis*	No	Not detected
4	76	F	No	No	4	Yes	*Escherichia coli*	No	Not detected
5	99	F	Yes	Yes (metabolic acidosis)	4	Yes	*Streptococcus agalactiae*	No	Not detected

**Table 5:** Changes in plasma ammonia levels and level of consciousness.

	Plasma ammonia levels (μg/dL) (Normal range 12–66 μg/dL)			Glasgow Coma Scale (score)		
Case	On admission	After 24 hours	After 5–7 days	On admission	After 24 hours	After 5–7 days
1	85	42	35	14	15	15
2	216	32	38	3	15	15
3	169	56	42	11	15	15
4	313	93	47	3	12	15
5	101	-	-	7	-	-

The case 5 patient died shortly after admission due to complicated aspiration pneumonia.

## Discussion

In this study, the incidence of hyperammonemia and disorders of consciousness caused by hyperammonemia was 8.3% in 60 targeted cases with urinary tract infections. There has been only prior report of a case of hyperammonemia caused by obstructive urinary tract infection and it is thus considered a rare pathologic condition [2], although we observed a relatively high incidence of this condition. Our study demonstrated that hyperammonemia caused by obstructive urinary tract infection is not a rare pathologic condition in elderly with poor performance status, of which the mean age was 76.3 ± 14.9 years; patients with ECOG-PS grade ≥2 accounted for 55.0% of these cases. Cases with hyperammonemia were significantly more likely to present with low GCS scores and urinary retention. When a case with a disorder of consciousness accompanies hyperammonemia, an obstructive urinary tract infection should be included in differential diagnosis.

Cases 1–4 with hyperammonemia accompanying obstructive urinary tract infections received antimicrobial therapy and an indwelling bladder catheter to relieve urinary retention, which resulted in rapid normalization of plasma ammonia levels and improved consciousness. The onset of hyperammonemia caused by urinary tract infections may occur as a result of elevated intravesical pressure due to dysuria caused by neurogenic bladder or prostatic hypertrophy. Then, urinary ammonia is absorbed by the vesical venous plexus and transferred to the systemic circulation through the inferior vena cava, instead of being transported via the liver through the internal iliac vein [2]. In addition, urease-producing bacteria in the bladder likely promote hyperammonemia [5,6].

When the urinary tract is infected with urease-producing bacteria, urease hydrolyzes urea in the urine to ammonium ions (NH^4+^), which elevate urinary pH. On the other hand, as ammonium ions become lipophilic ammonia (NH_3_) in the alkaline urine, it is easily transferred to the vesical venous plexus [2]. Hyperammonemia may develop due to urinary retention without infection by urease-producing bacteria [7]. *P*. *mirabilis*, *K*. *pneumonia*, and *Pseudomonas aeruginosa* are common urease-producing bacteria associated with urinary tract infections [8,9]. However, all bacteria causing hyperammonemia in this study were species that rarely produce urease [9] and urease activity was not detected in any of the cultured bacteria. The results of this study suggest that even without involvement of urease-producing bacteria, the fact that intravesical pressure is elevated and urinary ammonia is absorbed by the vesical venous plexus may cause hyperammonemia and accompanying disorders of consciousness. Therefore, along with antimicrobial administration, relief of obstruction is an effective treatment to reduce intravesical pressure and prevent absorption of urinary ammonia by the vesical venous plexus.

### Limitations

We targeted only hospitalized patients and observed the incidence of hyperammonemia in an elderly group with relatively poor performance status. Cases who had received antimicrobial therapy and exhibited cell counts <10^4^ CFU/mL at the time of urine testing were excluded; thus, the incidence in the general population with urinary tract infections is likely lower. This study included only five cases with hyperammonemia. To strengthen our conclusions, future studies should include a larger sample and a group of patients with hyperammonemia but who do not have a bladder catheter.

## Conclusions

The occurrence of hyperammonemia in urinary tract infections is not rare. The elevation of intravesical pressure due to urinary retention, followed by the absorption of urinary ammonia by the vesical venous plexus leads to hyperammonemia. Therefore, along with antimicrobial administration, relief of obstruction is important for the treatment of hyperammonemia being caused by this mechanism.
